# Dietary Intervention With α-Amylase Inhibitor in White Kidney Beans Added Yogurt Modulated Gut Microbiota to Adjust Blood Glucose in Mice

**DOI:** 10.3389/fnut.2021.664976

**Published:** 2021-10-12

**Authors:** Shenli Wang, Chongye Guo, Zhikai Xing, Meng Li, Haiying Yang, Yunting Zhang, Fazheng Ren, Lishui Chen, Shuangli Mi

**Affiliations:** ^1^Beijing Advanced Innovation Center for Food Nutrition and Human Health, College of Food Science and Nutrition Engineering, China Agricultural University, Beijing, China; ^2^Brand Food R&D Center, Nutrition & Health Research Institute (China Oil & Foodstuffs Corporation-NHRI), Beijing, China; ^3^Key Laboratory of Genomic and Precision Medicine, Beijing Institute of Genomics, Chinese Academy of Sciences/China National Center for Bioinformation, Beijing, China; ^4^University of Chinese Academy of Sciences, Beijing, China

**Keywords:** white kidney beans, α-amylase inhibitor, blood glucose, intestinal flora, 16S rRNA analysis

## Abstract

White kidney beans contain α-amylase inhibitors that can be used in diet for weight reduction. In this study, we investigated the potential of white kidney bean (*phaseolus vulgaris* L.) extract enriched in α-amylase inhibitor as a food additive in yogurt to regulate blood glucose in hyperglycemic animals. Five groups of C57BL/6J mice were fed for 8 weeks with standard chow diets, high-fat diets (HFD), or high-fat diets with supplement of α-amylase inhibitor in white kidney beans (*P. vulgaris* extract, PVE), yogurt (Y), and PVE added yogurt (YPVE), respectively. The HFD weakened glucose tolerance and caused insulin resistance in mice, and changed the characteristics of intestinal flora. The intervention of Y, PVE, and YPVE decreased blood glucose, insulin, hyperlipidemia, and inflammatory cytokine levels in mice fed with HFD. Moreover, the YPVE could regulate the components of host intestinal microbiota toward a healthy pattern, significantly increased the metabolic-related flora *Corynebacterium, Granulicatella*, and *Streptococcus*, while it decreased *Paraprevotella* and *Allobaculum*. Thus, YPVE markedly increased functions of “Amino Acid Metabolism,” “Energy Metabolism,” “Nucleotide Metabolism,” and declined functions of “Glycan Biosynthesis and Metabolism.” Consequently, YPVE could be developed as a new functional food because of its beneficial prebiotic properties in the metabolic syndrome.

## Introduction

White kidney bean is one species of *Phaseolus vulgaris* L., also known as common bean. It is consumed in considerable quantities around the world as a grain (1). It is a plant native to America, especially in the Andean and Mesoamerican regions (2). White kidney bean is also a good source of high proteins (16–33%), complex carbohydrates, and some vitamins and minerals. It can be incorporated into soup and stew. Some people decorticate and grind them into flour to make bakery products (3). In addition, the white kidney bean has been observed as a nutraceutical because it contains a variety of bioactive compounds, such as polyphenols, resistant starch, oligosaccharides, and bioactive peptides nutrients (4). White kidney bean is rich in potassium and magnesium, which are suitable for patients with hyperlipidemia, atherosclerosis, and heart disease (5, 6).

There is a growing recognition of the role of diet and other environmental factors in modulating the composition and metabolic activity of the human gut microbiota, which in turn can impact health (7). Abnormal changes of the intestinal microbiota could lead to metabolic syndrome-related disease: obesity (8), type 2 diabetes (9), and atherosclerosis (10). High-fat diet has been shown to reshape the gut microbiota composition, especially altering the proportion of *Bacteroidetes* and *Firmicutes*, contributing to the development of obesity and other metabolic disorders (11). Moreover, diet is an important element in the management of type 2 diabetes. Over the decades, microbiological studies proposed that intestinal flora could cause diabetes by regulating lipid metabolism to increase energy storage (12), producing short-chain fatty acids to mediate the release of intestinal hormones (13), and regulating bile acid metabolism to influence intestinal barrier function (14). Imbalance of intestinal flora has great influences on the host metabolism which eventually lead to occur and develop diabetes under a certain diet habit (15).

On the other hand, the white kidney bean is rich in phaseolin, a classical α-amylase inhibitor, and known as “starch blocker.” The α-amylase inhibitor is a natural bioactive component, which belongs to glycoside hydrolases and has anti-amylase activity in humans (16). Mounting evidence indicated that the white kidney beans extract enriched in α-amylase inhibitor exerts beneficial health effects on preventing hyperglycemic episodes and fat accumulation (17–20). It can reduce body weight gain and alleviate insulin resistance by changing the gut microbiota (11, 16). However, it remains unclear to what extents these gut microbial changes contribute to metabolic benefits.

In this study, we established a hyperglycemia model in middle-aged male mice on a high-fat diet (HFD) for the first time. Then, the experimental groups of these mice were administered orally *P. vulgaris* extract enriched in α-amylase inhibitor (PVE), yogurt (Y), and a mixture of yogurt with *P. vulgaris* extract enriched in α-amylase inhibitor (YPVE) for 8 weeks to detect the changes of their metabolic characteristics. We did 16S rRNA metagenome sequencing to analyze the regulation of PVE, Y, and YPVE on intestinal flora and the hypoglycemic mechanism, and further explore its potential mechanism.

## Materials and Methods

### Preparation and Analysis of White Kidney Bean Extract Enriched in Alpha-Amylase Inhibitors

The extract enriched of alpha-amylase inhibitors were purified from white kidney bean as described previously by Chi (21). The white kidney beans were first ground into a fine powder by sieving through an 80-mesh sieve. Two hundred grams of white kidney bean powder were added to distilled water with a ratio of 1:6 material and solvent, and agitated at 4°C to be fully dispersed. After an 8 h extraction, the supernatant was centrifuged at 4,000 *g* for 15 min at 25°C to obtain crude white kidney beans water-soluble extract. Then, the crude water-soluble extract was micro filtered with a 0.2 μm filter. According to our previous knowledge, most of the molecular weight of α-amylase inhibitors in common beans are in the range of 10–60 kDa. The extract was firstly filtered by using membrane of 50 kDa to remove large molecules. Then the filtrate supernatant was filtered again with a membrane of 10 kDa molecular mass, to remove small molecules. Finally, the retention supernatant with 10–50 kDa molecules was freeze-dried, pulverized, and used as the PVE in this study. The yield of the PVE was 4.56 g/100 g dried bean.

The protein, fat, and carbohydrates determination procedure was adopted from Association of Official Analytical Chemists (AOAC) (1999) (22). The alpha-amylase inhibitory activity of the extract was carried out according to a modified DNS (3,5-dinitrosalicylic acid) method (23). In brief, the PVE was dissolved in a sodium phosphate buffer (pH 6.9) containing 40 U/mL of porcine pancreatic α-amylase (Sigma-Aldrich, St. Louis, USA). The reaction solution was incubated in a water bath at 37°C for 30 min. Next, 400 μL of 2% starch solution as a substrate was added to 200 μL of each test solution and incubated at 37°C for 5 min. Then, 1,000 μL of DNS was added to the mixture and boiled for 10 min. After that, 150 μl of the mixed solution was transferred to a transparent-bottomed 96-well microplate and the absorbance at 540 nm was measured using microplate reader (Bio-TEK Instruments, Winooski, VT, USA). The activity of the α-amylase inhibitor (U/g) was defined as inhibition of α-amylase which cuts the starch chain into maltose (μmol) within 1 min.

### Animal Experiments

The animal experiments in this research were approved by the Ethics Committee of Beijing Institute of Genomics, Chinese Academy of Sciences with approval number of 2017H023. Sixty male C57BL/6J mice (12-month-old) were purchased from Sibeifu Laboratories (Beijing, China) and maintained on 12 h light/dark with access to food and water *ad libitum*. After a 1-week acclimatization period, mice were randomly divided into two groups; one group was provided with standard chow diet (*n* = 12) and the other group was fed with high fat diet (*n* = 48, 45% kcal from fat, D12451) for 12 weeks.

After 12 weeks, 48 of high fat fed mice were randomized again into four different experimental groups (12 animals per group) including HFD, PVE, Y, and YPVE groups. The HFD group received high fat diet only. According to dose conversion between animals and human (24), the PVE group received high fat diet with 180 mg/kg of PVE once per day, the Y group received high fat diet with plain yogurt (30 ml/kg), and the YPVE group was fed with a mixture of PVE with yogurt (180 mg/kg PVE+30 ml/kg yogurt). The nutrition fact of yogurt is fat content 3.1 g/100 g, total carbohydrates 8.5 g/100 g, protein 2.8 g/100 g, sodium 75 mg/100 g, and calcium 90 mg/100 g. The control group was fed with the standard chow diet. The supplementations of PVE, Y, and YPVE groups were administered *via* oral gavage.

Animals were treated for 8 weeks. Body weight and food intake were measured weekly throughout the study. At the end of experiment, mice were sacrificed to collect the blood. The blood was kept at room temperature for 30 min for coagulation. Serum samples were isolated by centrifugation at 1,000 *g* for 15 min at 4°C, then were snap-frozen and stored at −80°C for further analysis.

### Oral Glucose Tolerance Test (OGTT)

OGTT was performed after 8 weeks of treatment to test the function of pancreatic islet β cells and the glucose regulation ability of host. Mice with overnight fasting were orally administered two grams of glucose per kilogram body weight. Blood samples were collected from the tail vein at 0, 15, 30, 60, and 120 min after oral glucose loading. Blood glucose levels were measured at the indicated time points with a glucometer (Roche, ACCU-CHEK Active). The OGTT curve were made from glucose level vs. time. Then, Area under curve (AUC) of the OGTT curve were calculated according to the trapezoid method (25) to reflect the host body's metabolism of oral glucose.

### Insulin Resistance Measurement

The serum levels of insulin were determined by enzymatic methods using commercial kits (R&D Systems, Minneapolis, MN, USA). Insulin resistance (HOMA-IR) was measured by combining values of their fasting insulin and fasting glucose using a glucometer (Roche, ACCU-CHEK Active) with the following formula:


$$\begin{array}{l} \text{HOMA}-\text{IR}=[\text{fasting glucose }\left(\text{mM}\right)\\ \;\times \text{ fasting insulin }\left(\text{mUI}/\text{L}\right)]\;/\;22.5 \end{array}$$

### Blood Lipid Analysis

Before and after the establishment of the mice model and additive feeding testing, total triglycerides (TG), total cholesterol (TC), low density lipoprotein (LDL) cholesterol, high density lipoprotein (HDL) cholesterol of the hosts serum were recorded using an automatic biochemistry analyzer (ACCUTE TBA-40FR, Japan).

### Detection of Inflammatory Factors

The serum levels of insulin and circulating inflammatory cytokines, such as TNF-α and IL-1β, were determined by enzymatic methods using Mouse Quatikine ELISA kits (R&D Systems, Minneapolis, MN, USA).

### DNA Isolation and Library Construction

The frozen intestinal contents samples underwent fecal DNA extraction using QIAamp DNA stool mini kit (QIAGEN, cat#51504) according to the manufacturer's instructions. Concentration of the genomic DNAs was determined by spectrophotometry (Nanodrop ND1000; Thermo Scientific, USA) and its integrity was visually assessed by 1% agarose gel electrophoresis.

DNA libraries were constructed according to the manufacture's instruction of the NGS Fast DNA Library Prep Set for Illumina (Illumina, cat# CW2585M) and prepared with fragment length of ~300 bp and paired-end sequencing of the V4 hypervariable region of the bacterial 16S rRNA gene. Amplicon sequencing was performed on an Illumina MiSeq platform using MiSeq Reagent Kit (Illumina, cat#RH1021001). Paired-end reads were merged, demultiplexed, and analyzed by Quantitative Insights in to Microbial Ecology (QIIME) software (v2.2019.10) using a published bioinformatics pipeline (26). Sequences were assigned to OTUs with an open reference approach against the SILVA 16S rRNA reference database (release 128) clustered at 97% similarity. QIIME was used for the alpha diversity metrics to determine taxa richness (observed species) and faith's phylogenetic diversity (PD whole tree). The raw sequence data reported in this paper have been deposited in the Genome Sequence Archive (27) in BIG Data Center (28), Beijing Institute of Genomics, Chinese Academy of Sciences, under accession numbers CRA001468 and publicly accessible at http://bigd.big.ac.cn/gsa.

### Determination of Microbiota Composition and Function

Taxa with zero counts were normalized to a single count across all samples. Changes in taxon relative abundance were determined by calculating the log2 values of fold change in relative abundance against the anaerobically processed matching controls across all taxa. Taxon relative abundance average fold change was computed based on the inverse logarithm of the sum of log2 fold change divided by the number of donors in which the taxa was detected. Only bacterial taxa that were present (sequence count ≥ 2) in at least one comparison group and were present in at least 97% of samples were included in the analysis. Quantitative variables were expressed as mean value and standard deviation. Variables were compared using alpha and beta diversity analysis. Microbial metabolic function was predicted using Phylogenetic Investigation of Communities by Reconstruction of Unobserved States (PICRUSt) software. Then, dominant species were analyzed by calculating interactions among all of the microflora using Spearman's rank correlation coefficient. The threshold for significance was two-tailed, and *p*-value ≤ 0.05 was considered statistically significant. All of the statistical analyses were performed using R (version 3.5.2) software.

### Analysis of Correlation Between Microflora and Physiological Indexes

The correlation between the abundance of each bacterial genus and each blood glucose indicator was analyzed to obtain the Pearson correlation coefficient. The gut microbiota were ranked by the sum of their orders under each indicator according to the coefficients. The top 10 and bottom 10 bacterial genera were deemed glucose-related microbiota.

### Statistics

Data were expressed as means ± SEM of sample replicates. Unless otherwise specified, significance was determined using one-way ANOVA followed by Duncan's *post-hoc* tests. A *p*-value < 0.05 was considered to indicate statistical significance.

## Results

### Components and Amylase Inhibitory Activity of Phaseolus Vulgaris Extract

Firstly, the chemical components of the PVE were analyzed. We found out that the main constituent was protein of up to 75.4 ± 1.2 gram per 100 gram of the bean (g/100 g). Besides, carbohydrates were also detected with content of 14.5 ± 0.6 g/100 g. And the detection of fat showed that it was 2.8 ± 0.2 g/100 g. We also detected that the α-amylase inhibitor from our testing white kidney bean original in China was a structurally uniform glycoprotein with high content of protein, which is consistent with the previous studies in other white kidney beans (21). In our study, the α-amylase inhibitory activity against porcine pancreatic amylase of the extracts was 1,360 ± 30.2 U/g. The activity was slightly lower than that of previous report which was 1,840–5,777 U/g (29).

### Effects of Phaseolus Vulgaris Extract on Body Weight and Food Intake in HFD-Fed C57BL/6J Mice

**Figure 1:**
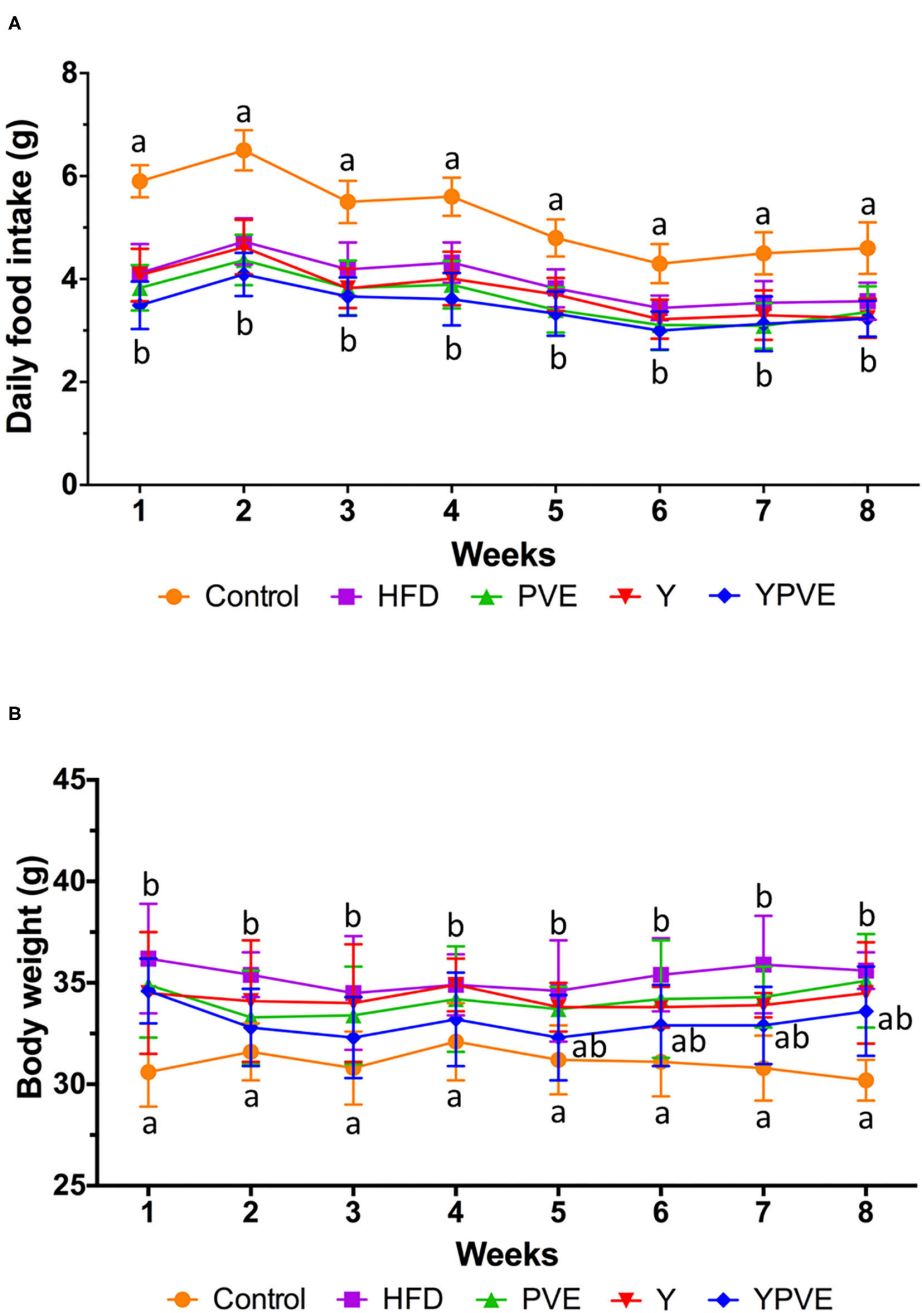
Daily food intake **(A)** and body weight **(B)** of C57BL/6J mice fed chow or high-fat diets for 8 weeks. Control: mice fed with a standard chow diet; HFD: mice fed with a high-fat diet; Y: mice fed with a high-fat diet with supplementation of yogurt; PVE: mice fed with a high-fat diet with supplementation of PVE. YPVE: mice fed with a high-fat diet with supplementation of PVE added yogurt. Values are presented as mean ± SEM, *n* = 12. Data not sharing a common superscript differ significantly among groups (*p* < 0.05) according to Duncan's *post-hoc* tests.

During the intervention, the daily food intake of HFD, Y, PVE, and YPVE groups were significantly lower than that of the control group (*p* < 0.05), and there was slight difference among the intervention groups (Figure 1A). The total intake of control, HFD, Y, PVE, and YPVE groups in 8 weeks were 292.32 ± 15.63 g, 222.16 ± 16.65 g, 210.06 ± 17.48 g, 202.17 ± 17.14 g, and 192.75 ± 18.97 g, respectively. As the caloric of the high-fat food was higher than that of the standard control food, the food intake of the control group was higher than that of the model and intervention groups. There was no significant difference among Y, PVE, and YPVE groups, which indicated that different supplementations had no effect on feed intake. Therefore, we believe that the additive of the extract of beans will not influence the appetite of subjects.

Furthermore, at the end of the model making period of the first 12 weeks (data not shown), the average weight of high-fat diet mice significantly heavier (36.22 ± 2.74 g) than chow-fed mice (30.51 ± 1.63 g). Under intervention, the body weight of each group was generally stable, suggesting that PVE and yogurt are generally safe as a food additive and would not affect the basic physical condition of subjects (Figure 1B).

### Effects of Phaseolus Vulgaris Extract on Blood Glucose, Glucose Tolerance, and Insulin Resistance in HFD-Fed C57BL/6J Mice

**Figure 2:**
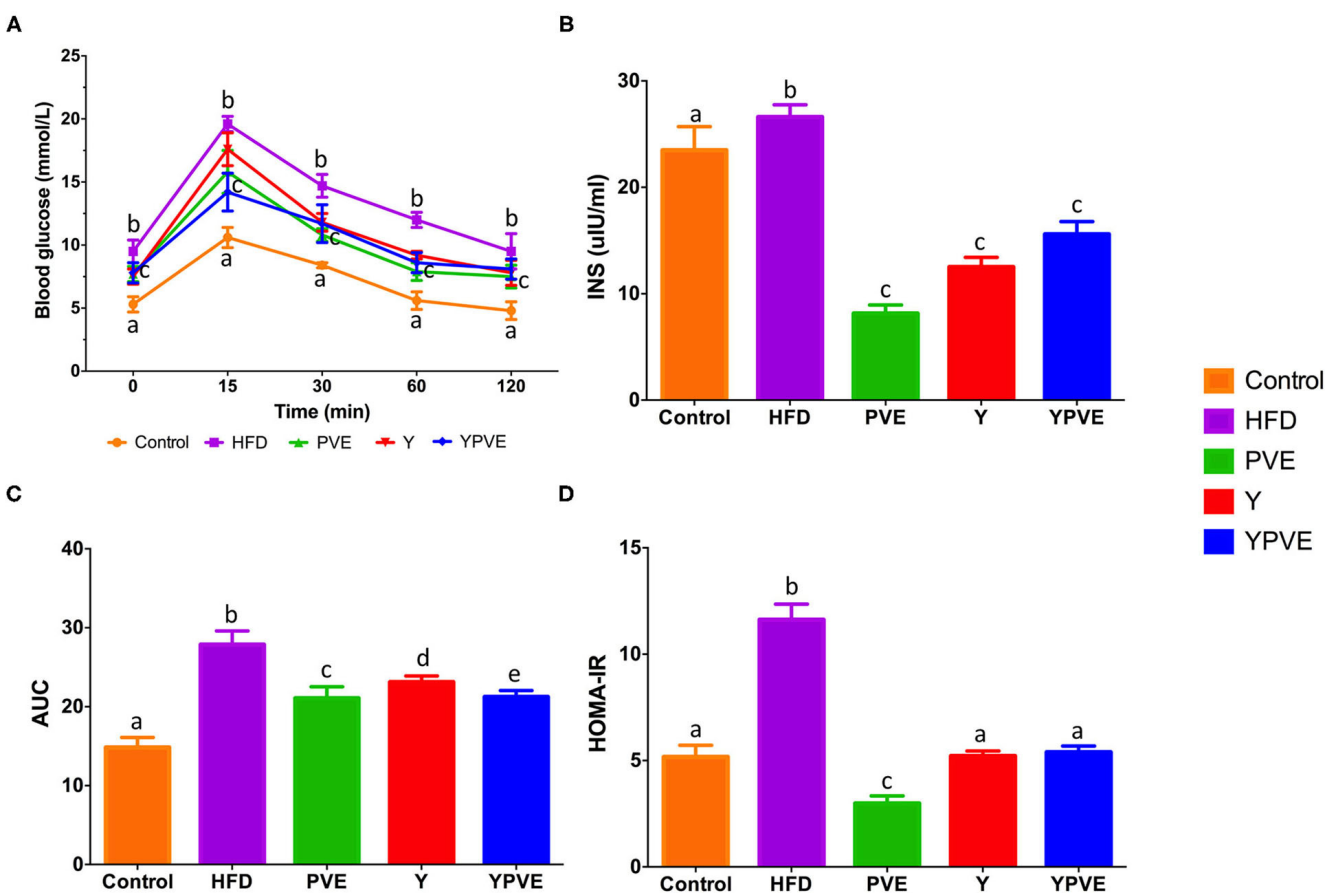
The oral glucose tolerance test (OGTT) **(A)**, AUC **(B)**, insulin concentration **(C)**, and HOMA-IR **(D)** in C57BL/6J mice fed with chow or high-fat diets for 8 weeks. Control: mice fed with a standard chow diet; HFD: mice fed with a high-fat diet; Y, mice fed with a high-fat diet with supplementation of yogurt; PVE: mice fed with a high-fat diet with supplementation of PVE. YPVE: mice fed with a high-fat diet with supplementation of PVE added yogurt. Values are presented as mean ± SEM, *n* = 8–12. Data not sharing a common superscript differ significantly among groups (*p* < 0.05) according to Duncan's *post-hoc* tests.

After 8 weeks on the corresponding diets, glucose tolerance and insulin sensitivity were tested. To investigate the effects of different diets on the prevention of postprandial hyperglycemia, OGTTs were performed at Week 8 (Figure 2A). Firstly, we recorded the fasting blood glucose at the beginning of the OGTTs (0 min) and found out that the blood glucose of mice in Y, PVE, and YPVE groups were significantly lower than HFD group (*p* < 0.05) (Figure 2A). After glucose loading, the blood glucose of HFD group always maintained a high level at 15, 30, 60, and 120 min. However, after administration of Y, PVE, and YPVE, the glucose levels of intervention groups decreased significantly at different time intervals, comparing with HFD group. The areas under the glucose tolerance curve (AUC) were showed 21.07 ± 0.78, 23.12 ± 1.46, 21.24 ± 0.81 for PVE, Y, YPVE, respectively, which were much lower than that of 27.9 ± 1.7 in the HFD group (*p* < 0.05) (Figure 2B). These indicators revealed that PVE, Y, and YPVE suppressed postprandial hyperglycemia in mice fed with high-fat diets.

After 8 weeks of high-fat diet, the serum insulin was tested. The HFD group was 26.62 ± 1.16 μlU/ml which is higher than control group (23.18 ± 1.90) (*p* < 0.05). The blood glucose was also high (9.5 ± 0.8 mmol/L) in HFD group. The serum insulin levels in the intervention groups (Y, PVE, and YPVE) were reduced by 53.1, 68.7, and 41.5, respectively comparing with HFD group (Figure 2C). The physiological indicators revealed that hyperinsulinemia was occurred with severe insulin resistance in HFD mice (Figure 2D). While in the intervention groups, the insulin resistances were suppressed and recovered to a similar level of control group in the YPVE group (Figure 2D). These results suggested that the intervention of white kidney bean extract and yogurt decreased the requirement of insulin to maintain comparable glucose concentration, significantly improved glucose tolerance, insulin sensitivity, and insulin resistance.

### Effects of Phaseolus Vulgaris Extract on Serum Lipid and Inflammation in HFD-Fed C57BL/6J Mice

**Figure 3:**
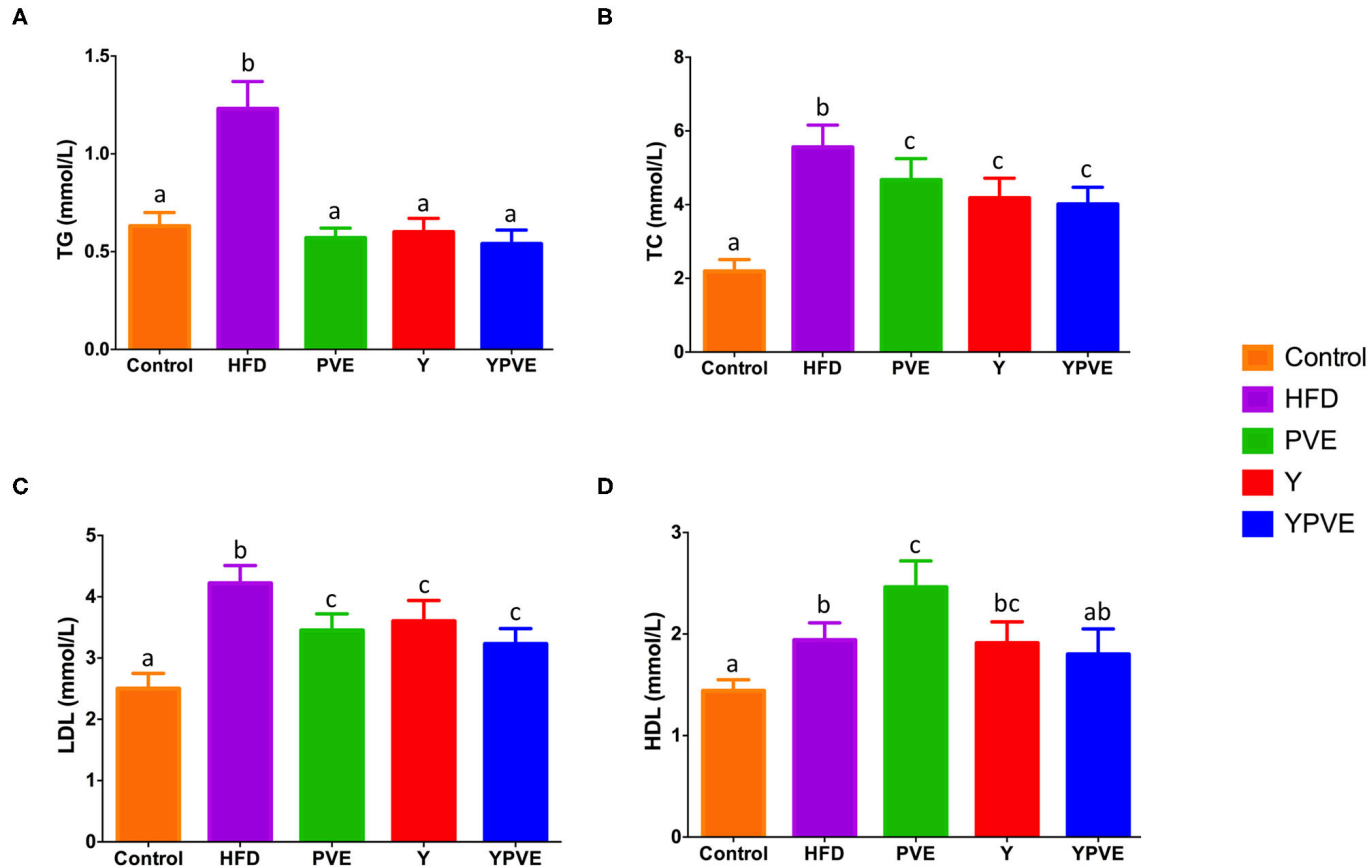
The serum TG **(A)**, TC **(B)**, LDL **(C)**, and HDL **(D)** level in C57BL/6J mice fed with chow or high-fat diets for 8 weeks. Control: mice fed with a standard chow diet; HFD: mice fed with a high-fat diet; Y: mice fed with a high-fat diet with supplementation of yogurt; PVE: mice fed with a high-fat diet with supplementation of PVE. YPVE: mice fed with a high-fat diet with supplementation of PVE added yogurt. Values are presented as mean ± SEM, *n* = 8–12. Data not sharing a common superscript differ significantly among groups (*p* < 0.05) according to Duncan's *post-hoc* tests.

TG, TC, LDL-C, and HDL-C levels were measured in the serum of mice at the end of feeding period (8 weeks). High-fat diet feeding induced an increasing in the serum TG, TC, LDL-C, and HDL-C. The serum levels of TG, TC, and LDL-C in HFD mice were increased by 95.1% (*p* < 0.01), 153.8% (*p* < 0.01), and 68.8% (*p* < 0.01), respectively (Figure 3). While treatment with different supplementations resulted in a significant decrease in serum concentrations of TG, TC, LDL-C. Comparing with HFD group, PVE administration increased the serum of HDL-C level significantly (2.46 ± 0.26 mmol/L vs. 1.94 ± 0.17 mmol/L, *p* < 0.05). All of these results could suggest that white kidney bean extract diet ameliorates the lipid profiles in serum of hyperglycemic mice.

**Figure 4:**
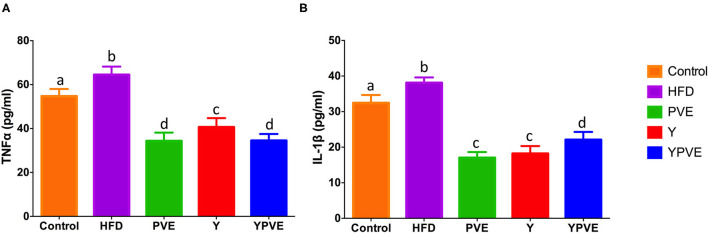
The serum TNF-α **(A)** and IL-1β **(B)** level in C57BL/6J mice fed with chow or high-fat diets for 8 weeks. Control: mice fed with a standard chow diet; HFD: mice fed with a high-fat diet; Y: mice fed with a high-fat diet with supplementation of yogurt; PVE: mice fed with a high-fat diet with supplementation of PVE. YPVE: mice fed high-fat diet with supplementation of PVE added yogurt. Values are presented as mean ± SEM, *n* = 8–12. Data not sharing a common superscript differ significantly among groups (*p* < 0.05) according to Duncan's *post-hoc* tests.

In this study, we also investigated the effects of different treatments on chronic inflammation in C57BL/6J mice (Figure 4). The data showed that HFD increased inflammatory cytokines (IL-1β and TNF-α) in C57BL/6J mice vs. that measured in control mice. However, TNF-α content were 34.33 ± 3.8 pg/ml, 40.63 ± 4.11 pg/ml, 34.54 ± 3 pg/ml for PVE, Y, YPVE groups, respectively, which decreased comparing with HFD group. Meanwhile, those values in IL-1β were also smaller in intervention mice than they were in hyperglycemic group mice (Figure 4). The administration of PVE, Y, and YPVE significantly decreased the serum level of IL-1β and the concentration of TNF-α, which illustrated that white kidney bean could improve the inflammatory response of subjects.

### High-Fat Diet Changes the Characteristics of Intestinal Flora Structures in Mice

To explore whether the intervention of PVE affected the characteristics of intestinal microbiota of experimental animals, we collected fecal samples from all of the subjects and extracted DNAs to profile the structural composition of the microbiota. In general, a total of 1068118 V416S rRNA sequences reads from the 30 samples with an average of 35,603 sequences reads for each sample (the minimum of one sample was 27,250 and the maximum was 61,850) were used for this project. The average length of sequence reads was 300 bp. Based on the results of the bacteria species counts, the richness was no significant change when the sample size was more than 20 which indicated that the sample size was enough for the sequencing analysis.

**Figure 5:**
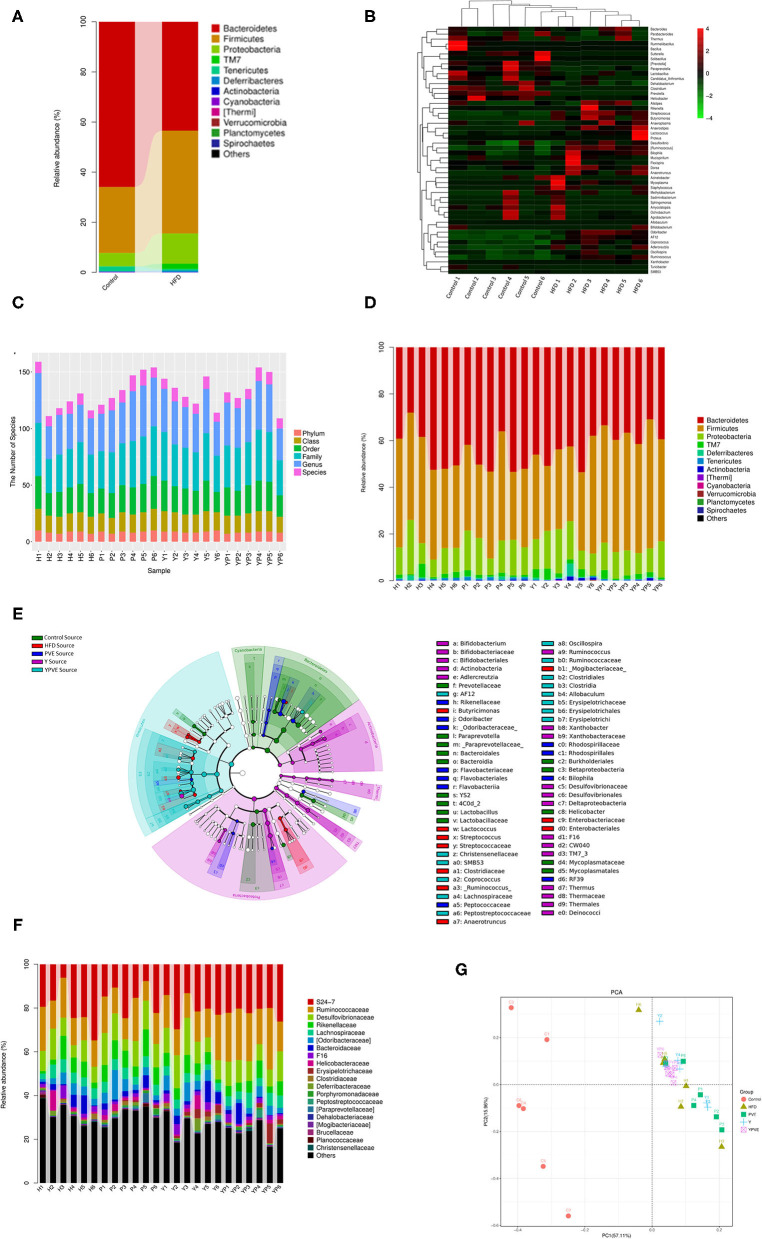
The diversity analysis of microbial composition of all sample groups. Control: mice fed with a standard chow diet; H/HFD: mice fed with a high-fat diet; Y: mice fed with a high-fat diet with supplementation of yogurt; P/PVE: mice fed with a high-fat diet with supplementation of PVE. YP/YPVE: mice fed high-fat diet with supplementation of PVE added yogurt. Values are presented as mean ± SEM, *n* = 12. **(A)** Stacked percent barchart of microbial population at phylum level in control and HFD groups. *Bacteroidetes* were decreased rapidly in HFD group comparing with control group. **(B)** Heatmap of combination cluster analysis of microbial composition in genus level. Red represents higher abundance, while green represents lower abundance. Most of the increased bacteria in HFD group were harmful, including pathogenicity bacteria *Streptococcus* and *Proteus*, while, probiotics such as *Prevotella, Lactobacillus* and *Bacillus* reduced in HFD samples. **(C)** Stacked percent barchart of microbiota at different classification levels in different intervention groups. The diversities of intestinal microbial species in each group were generally no significant different. **(D)** Stacked percent barchart of microbiota at genus level in different intervention groups. The percentage of *Firmicutes* in YPVE group was higher than that in HFD group. While, comparing with other groups, the percentage of *Bacteroides* and *Proteobacteria* in YPVE group decreased. **(E)** Bacteria classification hierarchy tree based on mouse group differences displayed by cladogram. The most significantly changed bacteria were appeared in *Clostridia* and *Bacteroidetes*, such as *Coprococcus, Allobaculum, Oscillospira*, and *AF12*. **(F)** Stacked percent barchart of microbiota at genus level in different intervention groups. **(G)** two-dimensional sorting diagram of samples for PCA. Each point represents a sample, and different color belongs to different groups. The closer the distance between two points, the higher the similarity of microbial communities between the two samples, the smaller the differences. The samples in YPVE group were approaching into the same quadrant, which is much closer than the rest of groups.

Based on the composition and structure of intestinal flora from control group and HFD group, we noticed that the high-fat diet can lead to changes in intestinal bacteria. At the phylum level, *Bacteroidetes* were decreased rapidly in HFD group comparing with control group after 12 weeks feeding (Figure 5A). It was reported as a type of weighted and healthy related bacteria, while has been checked as a positive correlation bacteria with vegetarianism and lean persons (30). Furthermore, *Firmicutes* and *Proteobacteria* were both noticeably increased in HFD group over controls (Figure 5A). Generally, *Firmicutes* is another type of bacteria which is positively related to obesity of their host (31). Besides, *Proteobacteria* is the largest of bacteria, containing many pathogenic bacteria, such as *E. coli, Salmonella, Vibrio cholera*, etc. (32). Therefore, the rise of these two bacteria phyla will increase the risk of illness for their host. At the genus level, the control and HFD samples were divided clearly by the significantly changed bacteria (Figure 5B). As shown in Figure 5B, most of the increasing bacteria in HFD group were harmful, including pathogenicity bacteria *Streptococcus* and *Proteus* etc. Meanwhile, most of the probiotics were reduced in HFD samples, such as *Prevotella, Lactobacillus*, and *Bacillus*. All the above revealed that diet can change the characteristics of intestinal flora, and the high-fat diet leads to an unhealthy characteristic.

### Microbiota Composition Developed Toward a Healthy Pattern by Dietary Intervention of Yogurt With White Kidney Beans Additive

Rely on diversity analysis, we observed that the diversities of intestinal microbial species in each group were generally not significantly different (Figure 5C). At the phylum level, the abundance of *Firmicutes* in YPVE group remained higher level than HFD group. Meanwhile, the *Bacteroidetes* and *Proteobacteria* in YPVE group was declined comparing to the rest of groups (Figure 5D). These results reminded us that, as a food additive of compound formula, the YPVE was leading to a healthy direction on the regulation of intestinal flora. In addition, the classification hierarchy tree (Figure 5E) showed that in YPVE group, the most significantly changed bacteria appeared in *Clostridia* and *Bacteroidetes*, such as *Coprococcus, Allobaculum, Oscillospira*, and *AF12*.

Furthermore, we found that YPVE group showed more stable composing and more positive changes in the intestinal flora (Figure 5F). For instance, *S24-7* appears to negatively correlate to type 2 diabetes (33), and it increased in YPVE group much higher than other groups. The abundance of *Ruminococcaceae* was raised in YPVE group, but was the lowest in HFD group. It could cause lipid metabolism of HFD mice to decrease (34). Also, reported as opportunistic pathogens (31), *Desulfovibrionaceae* was less in YPVE group than those in other intervention groups. Summing up, we believed that YPVE intervention led to microbiota composition developing toward a healthy pattern.

Moreover, we utilized PCA analysis to discover the similarity of main bacteria between the samples. As Figure 5G showed, the samples in YPVE group were approaching into the same quadrant, which is much closer than the rest of groups. It indicated that YPVE reduced the individual difference in a short time and made the intestinal flora more consistent between individuals. Therefore, YPVE tends to have more forceful regulation ability than single formula additive.

**Figure 6:**
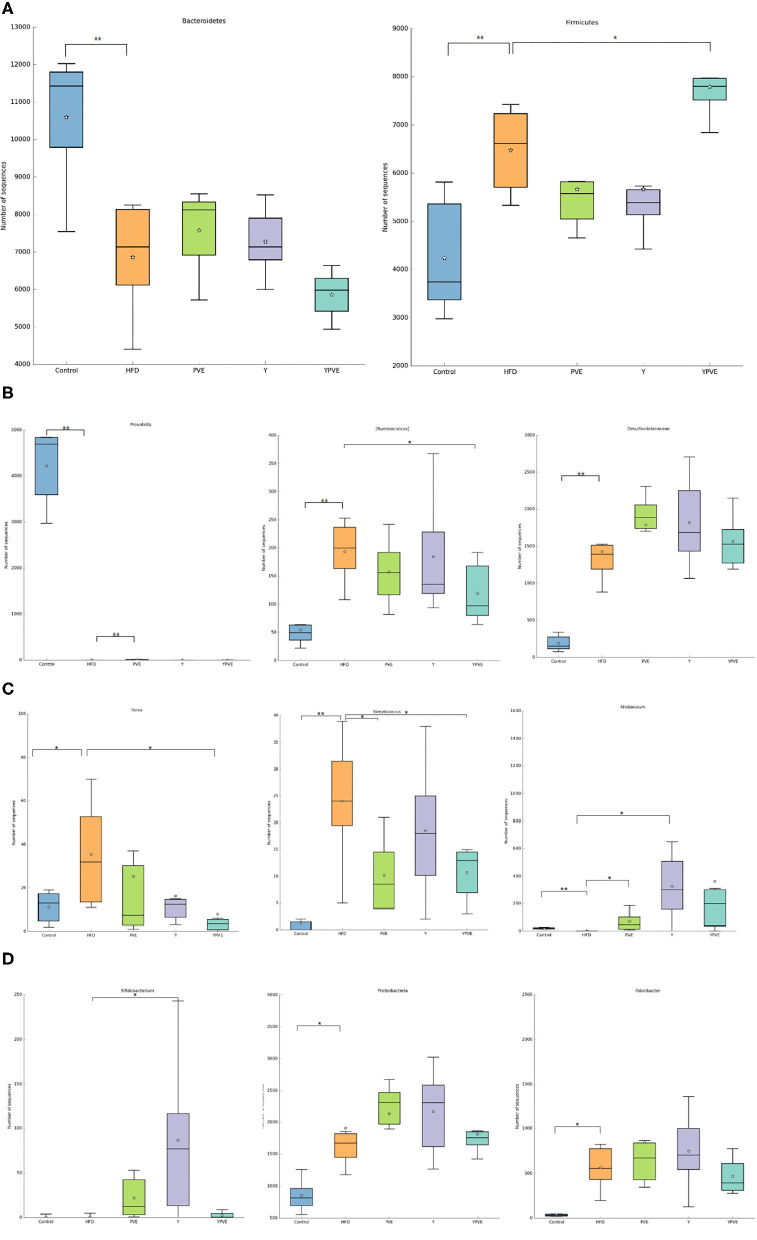
The abundance of specific bacteria in HFD, Y, PVE, and YPVE groups. HFD: mice fed with a high-fat diet; Y: mice fed with a high-fat diet with supplementation of yogurt; PVE: mice fed with a high-fat diet with supplementation of PVE. YPVE: mice fed high-fat diet with supplementation of PVE added yogurt. Values are presented as mean ± SEM, *n* = 12. **(A)** Abundance of *Bacteroides* (left) and *Firmicutes* (right) in four sample groups at phylum. Y and PVE supplement the abundance of *Bacteroides*. However, YPVE reduce the abundance and proportion of *Bacteroides*. In the right panel, YPVE increased the abundance of *Firmicutes*, contrary to the other two groups (Y and PVE). **(B)** Abundances of *Prevotella* (left), *Ruminococcu*s (middle), and *Desulfovibrionaceae* (right) in each group at the genus level. PVE group increased the abundance of *Prevotella* in a small amount compared with HFD group, but the other two groups were not able to supplement *Prevotella*. YPVE group reduced *Ruminococcus*. The three intervention groups had no significant effect on the genus and family of *Desulfovibrionaceae*. **(C)** Abundance of *Dorea* (left), *Streptococcu*s (middle), and *Allobaculum* (right) in each group. The intervention YPVE reduced the increase of *Dorea* and *Streptococcus* compared with HFD group. PVE reduced the increase of *Streptococcus* by HFD. All three intervention groups increased the abundance of *Allobaculum*. **(D)** Abundance of *Bifidobacterium* (left), *Proteobacteria* (middle), and *Odoribacter* (right) in each group. Y group increased the abundance of *Proteobacteria*. Difference between groups was assessed using one-way ANOVA followed by Duncan's *post-hoc* tests: *p < 0.05, **p < 0.01.

Through the analysis of species composition, we found that the three groups of diet intervention had a certain impact on the intestinal flora of mice, weakened the impact of high-fat diet on the intestinal flora, and made the intestinal flora develop toward a healthy direction. There was an abundance of *Bacteroides* and *Firmicutes* in four sample groups at phylum. Y and PVE supplement the abundance of *Bacteroides*. However, YPVE reduce the abundance and proportion of *Bacteroides*. In the right panel, YPVE increased the abundance of *Firmicutes*, contrary to Y and PVE groups (Figure 6A). At the genus level, PVE group increased the abundance of *Prevotella* in a small amount compared with HFD group, but the other two groups were not able to supplement *Prevotella* due to the high-fat diet. YPVE group reduced *Ruminococcus*. The three intervention groups had no significant effect on the genus and family of *Desulfovibrionaceae* (Figure 6B). The intervention YPVE reduced the increase of *Dorea* and *Streptococcus* compared with the HFD group. PVE reduced the increase of *Streptococcus* by HFD. As known, *Streptococcus* contained many pathogenic bacteria. All three intervention groups increased the abundance of *Allobaculum* (Figure 6C). There are few studies on the relationship between *Allobaculum* and diabetes, suggesting that *Allobaculum* may grow more easily under the health condition. The addition of yogurt in Y group greatly increased the abundance of *Bifidobacteria*, while the increase of *Bifidobacteria* in PVE and YPVE was less than that in Y (Figure 6D). It has been proved that there is an increase in the number of *Akkermansia* bacteria in Y group. The addition of YPVE reduced the abundance of *Proteobacteria* and *Odoribacter*, which contain multiple pathogenic bacteria, indicating that YPVE is more conducive to inhibiting opportunistic pathogens and maintaining the overall balance of intestinal flora.

### Phaseolus Vulgaris Extract Added Yogurt Promoting the Functions of Intestinal Flora to Be Instrumental in Host Health

**Figure 7:**
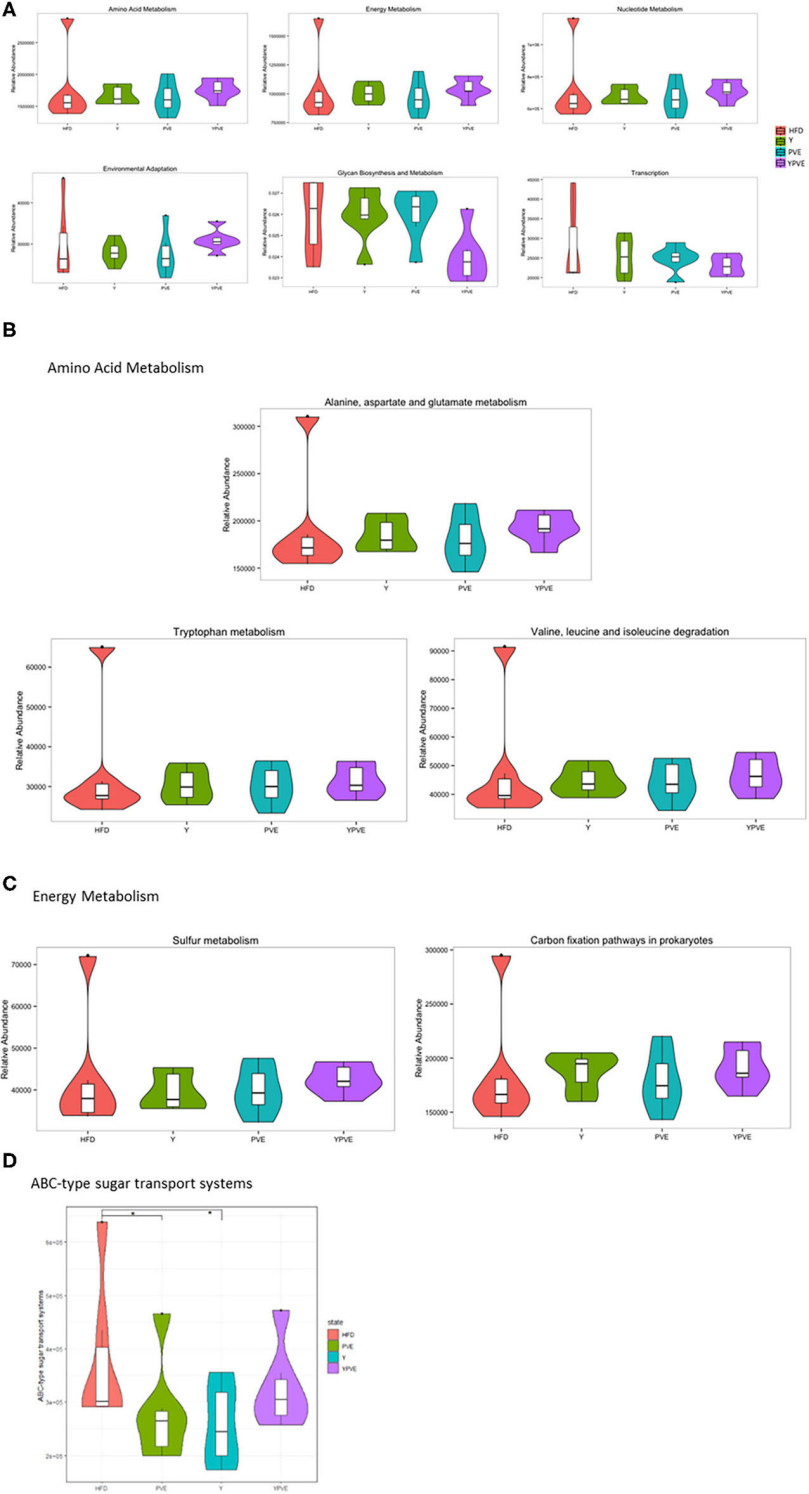
Comparative analysis of functional relative abundance of intestinal flora in different mice groups. Control: mice fed with a standard chow diet; HFD: mice fed with a high-fat diet; Y: mice fed with a high-fat diet with supplementation of yogurt; PVE: mice fed with a high-fat diet with supplementation of PVE. YPVE: mice fed high-fat diet with supplementation of PVE added yogurt. Values are presented as mean ± SEM, *n* = 12. **(A)** The top six bacterial functional catalogs that vary between groups. **(B)** The sub-functions in Amino Acid Metabolism pathway that differ between groups. **(C)** The sub-functions in Energy Metabolism pathway that differ between groups. **(D)** The functional abundance of a sub-function “ABC-type sugar transport systems” increased significantly in PVE and Y groups compared with HFD. Significance was assessed using one-way ANOVA followed by Duncan's *post-hoc* tests: *p < 0.05.

Through KEGG functional prediction and abundance calculation, we found out that several essential functions of gut microbiota varied in different groups, especially between YPVE and HFD group. The most obvious changes occurred in metabolic functions, including increased functions of “Amino Acid Metabolism,” “Energy Metabolism,” “Nucleotide Metabolism,” and declined functions of “Glycan Biosynthesis and Metabolism” (Figure 7A). Furthermore, the functions of “Environmental Adaptation” and “Transcription” were exhibited a rising and falling trend, respectively (Figure 7A).

We further checked the abundances for all of the sub-functions in each of the “Metabolism” functions. As shown in Figure 7B, the most increasing sub-function was “alanine, aspartate and glutamate metabolism” in “Amino Acid Metabolism” after comparing YPVE with HFD group. In clinical studies, higher alanine metabolism improves the metabolism of triglyceride, which may beneficially affect the cardiometabolic health status of men with type 2 diabetes (35). In addition, “sulfur metabolism” and “carbon fixation pathways in prokaryotes” as parts of “Energy Metabolism” were improved in YPVE group (Figure 7C). These two functions positively associated with the increasing of anaerobic bacteria which known to be probiotics (36). In addition, the functional abundance of a sub-function “ABC-type sugar transport systems” increased significantly in PVE and Y groups compared with HFD (*p* < 0.05), which proved that the utilization of polysaccharides in food was increased eventually (Figure 7D). In general, YPVE could promote the functions of intestinal flora to be instrumental in the host health.

### Intestinal Flora Related to Glucose Metabolism

**Figure 8:**
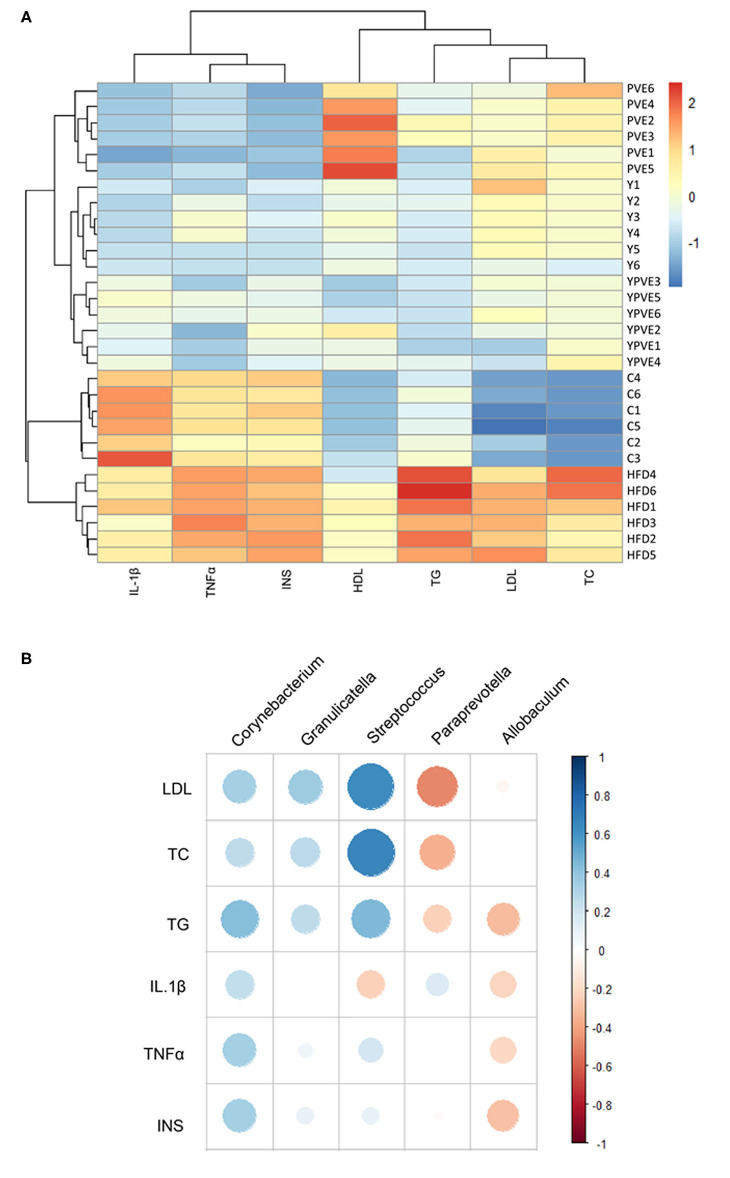
The clustering for all sample groups and correlation analysis between intestinal flora and physiological indictors. Control: mice fed with a standard chow diet; HFD: mice fed with a high-fat diet; Y: mice fed with a high-fat diet with supplementation of yogurt; PVE: mice fed with a high-fat diet with supplementation of PVE. YPVE: mice fed high-fat diet with supplementation of PVE added yogurt. Values are presented as mean ± SEM, *n* = 12. **(A)** The clustering result for all samples based on all of the blood indictors, including IL1β, TNFα, INS, HDL, TG, LDL, and TC. **(B)** A correlation analysis between intestinal flora and physiological indictors using different groups. All of the intestinal bacteria in the groups of this chart were compared with those in control groups first. Orange represents positive correlation, while blue represents negative correlation.

Based on all the indicators, the mice could cluster into different groups which were consistent with their dietary treatments (Figure 8A). Since each group of mice have their specific gut microflora, the microflora could be associated with physical indicators. Through the correlation analysis between glucose related physiological indicators and the abundance of intestinal flora, we were able to gain the microbiota which was associated with blood glucose regulation. Comparing the intestinal microflora between intervention groups and HFD group, five of the glucose related microflora had simultaneously significant changes, containing three bacteria that positively related to the indicators (*Corynebacterium, Granulicatella*, and *Streptococcus*) and two bacteria that negatively related to the indicators (*Paraprevotella* and *Allobaculum*) (Figure 8B). These results told us that the food additives in this study could effectively reduce the metabolism of glucose in hyperinsulinemia mice by altering the composition of intestinal flora, especially by induction the change of *Corynebacterium, Granulicatella, Streptococcus, Paraprevotella*, and *Allobaculum*.

## Discussion

In recent years, due to its short growth cycle, economic efficiency, and consistent with the biochemical and pathological features of human diabetes, rodent model animals were common models used for investigating the effect of diet on metabolic risk factors, such as lipid profiles and plasma glucose levels. High-fat and high-fructose diets as important environmental factors can induce insulin resistance in mice, which has been recognized widely. Middle-aged mice appear to be more susceptible to developing insulin resistance while gaining more weight under HFD feeding compared with young animals (37). In this study, we used 12 weeks high-fat fed diet for 52-week-old C57BL/6J mice as an animal model, followed by 8 weeks of intervention additive feeding. This study reported for the first time the effects of white kidney beans extract, yogurt, and white kidney extract yogurt on glucose regulation and modulation of gut microbiota in high-fat fed mice, respectively. Dietary interventions have played an important role for preventing the metabolic syndrome disease. In recent decades, growing evidences indicated that consumption of plant-derived foods is possibly inverse correlated with several features of the metabolic syndrome, thus reducing the risk of type 2 diabetes and cardiovascular disease. Previous evidences demonstrated that PVE have potential effects on human health, and possess anti-oxidant (38), anti-carcinogenic (39), anti-inflammation (40), anti-obesity (16, 41, 42), and anti-diabetes and cardioprotective properties (16, 43). The potential action site of P. *vulgaris* on hyperglycaemia and body weight have been proposed to the presence of α-amylase inhibitor. α-amylase inhibitors isolated from white kidney beans are glycoproteins with an oligomeric structure comprised of identical peptides. The C-terminal residue was mostly serine, alanine and tyrosine, and N-terminal having an oligosaccharide moiety linking a glycosylation site (44). The amino acid composition of the glycoprotein was mainly aspartic acid, glutamic acid, threonine, and serine. The monosaccharide of the sugar chain was composed of mannose, glucose, galactose, and xylose. The glycopeptide bond type of sugar and protein was oxygen glycopeptide bond. The sugar chain or protein chain alone is not biologically active unless they are combined to form a complete glycoprotein (45). α-amylase inhibitor interacts with the glycoside of the saliva and pancreatic α-amylase in the digestive tract through non-competitive inhibition, reducing its enzyme activity. Starch digestion was prevented by α-amylase inhibitors, which completely block access to the active site of the alpha-amylase enzyme. Furthermore, the undigested starches can be fermented by the colonic bacteria to produce carbon dioxide, short-chain fatty acids, and influence the intestinal ecosystem of host. Therefore, we believe that the mechanism behind the improving effects of PVE relies on the activity of the a-amylase-inhibiting.

Insulin resistance, hyperglycemia, and hyperlipidemia are the major important markers of type 2 diabetes (46). Hyperglycemia is marked in the postprandial state, due to intestinal absorption of the glucose generated from a meal, which occurs across the epithelial enterocytic cells of the small intestine, involving glucose transporters. In this study, the OGTT test and AUC showed progressively reduced glucose levels in mice supplemented with PVE yogurt after glucose administration, suggesting an improved glucose tolerance. The fasting serum insulin level and insulin resistance were also reduced in the intervention groups, suggesting the hyperinsulinemia-improving effect.

Insulin resistance usually causes abnormal lipid metabolism in body, since insulin can promote lipoprotein decomposition. When the body produces insulin resistance, the serum levels of TG, low-density lipoprotein cholesterol, and very low-density lipoprotein cholesterol are significantly increased. Therefore, diabetes is not only a disorder of glucose metabolism but also a disorder of lipid metabolism (47). In this study, insulin levels and blood lipids were tested in the intervention groups and the control group. The results showed that the serum insulin levels in the model group were slightly higher than those in the control group, but there was a significant difference. It is speculated that a long time of high fat diet specifically destroys insulin-producing islet β cells and inhibits the ability to secrete high level of insulin in the body. However, as the blood glucose level of the model group increases, insulin secretion can be stimulated. The increased level of serum insulin should be the result by effects of the above factors. In addition, our study also found that serum TC, TG, LDL levels in the HFD group were significantly higher than those in normal mice, consistent with previous studies [11]. HDL levels in PVE and Y group were significantly higher than normal mice, and there was no significant difference in HDL concentrations between the two groups of mice. In the intervention groups, the PVE, Y, and PVE with yogurt significantly decreased mice cholesterol, triglyceride, LDL cholesterol, and increased the high-density lipoprotein cholesterol, reduced lipid metabolism disorder, and gradually restored the blood lipid level of glycemic mice to the normal state. These results suggested that PVE as food additives in yogurt have potentials for anti-diabetic activity and play a positive role in dyslipidemia.

Impaired glucose tolerance and insulin resistance are closely associated with chronic inflammation characterized by abnormal cytokine production (48). Treatment of PVE, Y, and YPVE reduced the production of several inflammatory cytokines, including TNF-α and IL-1β. TNF-α and IL-1β play key roles in the obesity, T2D, and metabolic disorders, disrupting the insulin and lipid signaling pathways, thereby influencing insulin sensitivity and lipid metabolism. In humans, the circulating concentration of TNF-α is strongly associated with impaired glucose tolerance, enhanced insulin resistance, and increased T2D risk (49, 50). TNF-α is a mediator of insulin resistance through blocking the action of insulin (51). It has been reported that interactions between diet and enteric bacteria were necessary for inducing inflammatory changes in the intestine and to impact diet-induced obesity and insulin resistance (52). Therefore, our observations suggest that PVE treatment may be a novel strategy against intestinal inflammation and the prevention of the metabolic syndrome in high-fat diet mice.

Previous studies indicated a triangle of diet-gut microbiota-host metabolism (53, 54). It is widely accepted that beneficial bacteria produce essential nutrients, including vitamins and organic acids, and the gut epithelium and vital organs such as liver will absorb these nutrients to improve the host healthy (53). On the other hand, gut flora influence inflammatory responses and the gut-brain axis resulting in the weight-gain and adiposity through several inter-connected pathways, containing energy harvest and production of microbial metabolites (54). Therefore, the α-amylase inhibitor from white kidney beans may exert prebiotic effects and reshape the gut microbiota with benefits to the host.

To verify this deduction, the alpha diversity and beta diversity of gut microbiota were analyzed in our study. We firstly noticed the richness and abundance of microbial species in intestinal flora of YPVE mice feeding with white kidney bean yogurt. According to alpha diversity analysis, the richness of bacteria was significantly increased in YPVE group comparing with HFD group, which indicates that the intestinal bacteria of the YPVE mice could better maintain their dynamic balance and were not easily affected by external factors, so that the metabolic function of YPVE mice was gradually restored even in high-fat diet feeding mode. This phenomenon has also been confirmed in the detection of physiological indicators (Figure 2). Beta diversity analysis also found that only YPVE mice could be gathered well in the same quadrant, revealing that the convergence effects of the combined additive on intestinal microorganisms were stable and rapid, rather than random. Furthermore, when comparing the differences in the number of dominant flora among the testing groups, it was observed that the most varied genus including *Oscillospira, Prevotella, Odoribacter, Bacteroides*, and *Ruminococcus* were all related with the host metabolism (55–61). Based on our results and the results of other studies, we can infer that the white kidney bean extract regulates blood glucose by changing these bacteria and making the metabolism of the host healthy.

In the study of the association between functions of gut microbiota and T2D, we noticed that previous study provided that individual amino acid levels of their intestinal flora were significantly associated with insulin sensitivity and insulin secretion (62). Similarly, “Energy Metabolism” also affects insulin sensitivity (63). Moreover, changing of “Nucleotide Metabolism” could reduce the metabolism of purine base by intestinal bacteria, and thus improve the ability of host cells to absorb nutrients and oxygen by reducing the production of acid fatigue substances in the body, such as lactic acid (64). On the contrary, the declining of “Glycan Biosynthesis and Metabolism” function indicated that the ability of bacteria to metabolize polysaccharides into monosaccharides decreased, which could effectively reduce the utilization of polysaccharides in food. All the previous studies are consistent with our data, which made us find that yogurt with the extract of white kidney beans could rise the number of anaerobic bacteria and decline the absorption and metabolism of polysaccharides by the intestinal flora, thus ultimately regulated the host blood glucose.

This blood glucose regulation function of YPVE was confirmed by the follow-up association analysis between physical data and microbiota of all samples and five bacteria in genus showed the most closely related to glucose metabolism, including *Corynebacterium, Granulicatella, Streptococcus, Paraprevotella*, and *Allobaculum*. Previous studies have shown that these five microflorae are related to glucose metabolism. Chen et al. pointed out that *Corynebacterium* showed increasing in Type 2 diabetes (T2D) samples, contrary to *Allobaculum* which was decreased in T2D samples comparing with health samples (65). *Streptococcus* and *Granulicatella* were observed to be related to glycosylation process (66). Moreover, *Streptococcus* can improve glycemic parameters in T2D mice (67) and has been used as probiotics to help prevent insulin resistance (68). Also, *Paraprevotella* is related to glucose tolerance, which shows the higher *Paraprevotella*, the worse in tolerance (69). The positive correlation appeared strongly between bacteria (*Corynebacterium, Granulicatella*, and *Streptococcus*) and the host blood glucose and lipids, while the other two bacteria (*Paraprevotella, Allobaculum*) showed the opposite results. These five bacteria were all regulated by the diet types, and the abundance in YPVE group approached the healthy level.

## Conclusion

In summary, YPVE can effectively adjust blood glucose, restore the blood lipid and inflammatory cytokines level of glycemic mice to the normal state, thus improve the intestinal health of the host. It significantly increased the metabolic-related flora *Corynebacterium, Granulicatella*, and *Streptococcus*, while it decreased *Paraprevotella* and *Allobaculum*. This change in intestinal microbiota may reduce the metabolism function of the host, decrease the production of some substances such as lactic acid, and decline the utilization rate of polysaccharides in the host intestine. Therefore, YPVE modulated gut microbiota to adjust blood glucose and could be developed as a new functional food with beneficial prebiotic effects in the metabolic syndrome.

## Data Availability Statement

Raw sequences have been deposited to Genome Sequence Archive (GSA) database (https://bigd.big.ac.cn/gsa/) with the access number of CRA001468.

## Ethics Statement

The animal study was reviewed and approved by Beijing Institute of Genomics, Chinese Academy of Sciences/China National Center for Bioinformation No. 2017H023.

## Author Contributions

SM and LC conceived the project and designed and supervised the experiments. SW prepared α-amylase inhibitor from white kidney beans, designed the experiments, made animal models, performed the dietary intervention experiments, and analyzed the physiological data. CG and ZX analyzed and visualized the 16S rRNA sequencing data. CG, ZX, ML, and YZ prepared stool DNAs for sequencing. HY and FR contributed to mice fed and operated instruments. SM, SW, and CG wrote the manuscript. All authors contributed to the article and approved the submitted version.

## Funding

This work was supported by Beijing Municipal Science and Technology Project (Grant No. D171100008017001) and the Strategic Priority Research Program of Chinese Academy of Sciences (Grant No. XDB38030400).

## Conflict of Interest

The authors declare that the research was conducted in the absence of any commercial or financial relationships that could be construed as a potential conflict of interest.

## Publisher's Note

All claims expressed in this article are solely those of the authors and do not necessarily represent those of their affiliated organizations, or those of the publisher, the editors and the reviewers. Any product that may be evaluated in this article, or claim that may be made by its manufacturer, is not guaranteed or endorsed by the publisher.
